# Supplementary figures and images for: Comparing the effectiveness of environmental DNA and camera traps for surveying American mink (Neogale vison) in northeastern Indiana
Source: PLoS One. 2024 Sep 23;19(9):e0310888. doi: 10.1371/journal.pone.0310888 (PMC11419345; doi:10.1371/journal.pone.0310888)

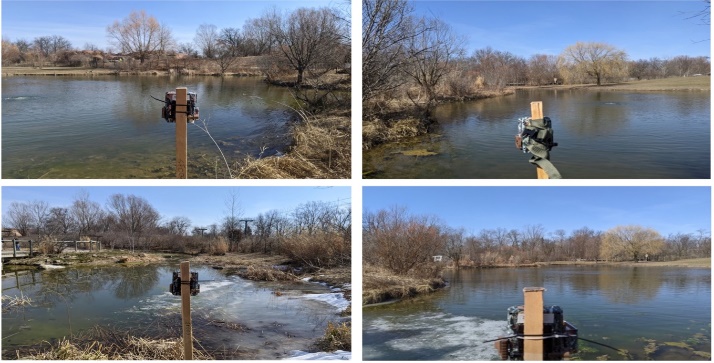
S2 Fig.

Supplement: S2 Fig — Site was located in the African exhibit. (DOCX) [file pone.0310888.s002.docx]

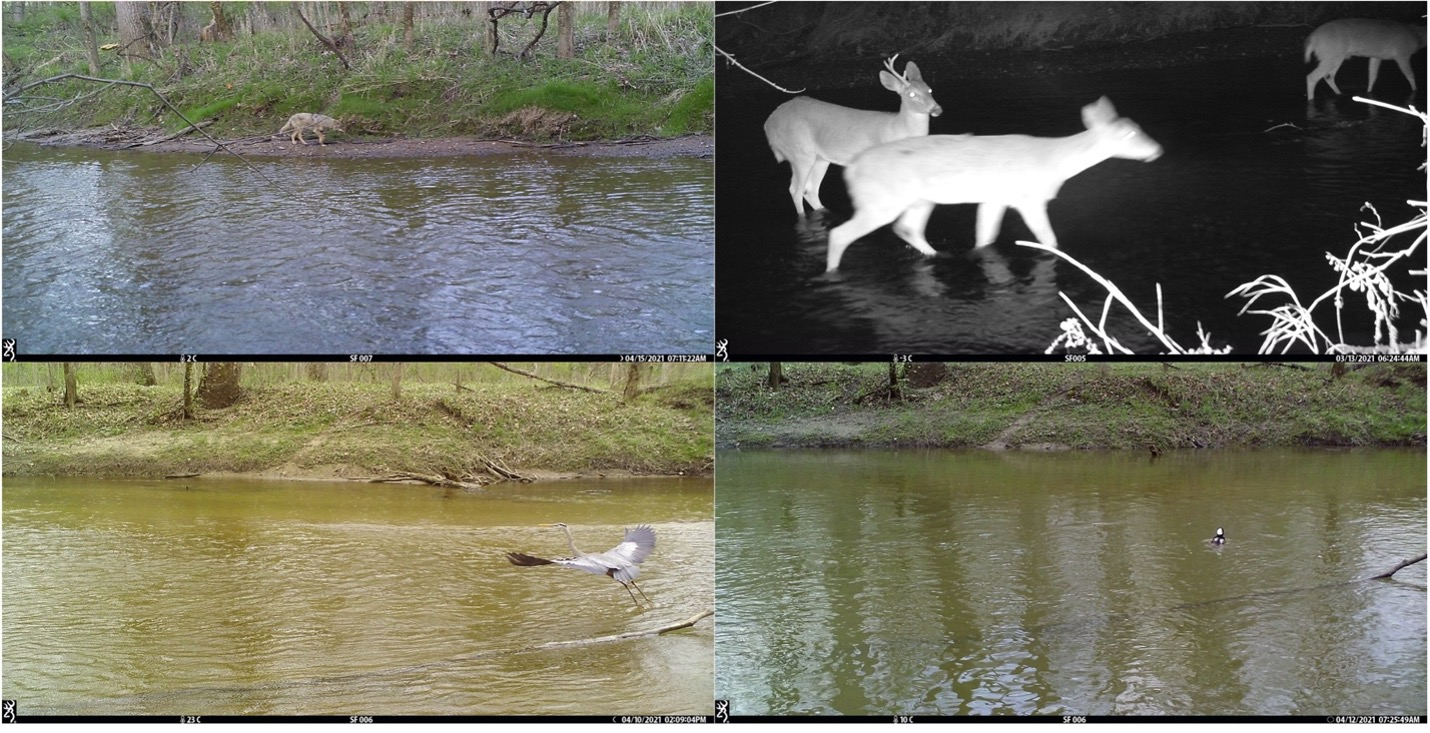

Supplement: S3 Fig — Includes photos of a coyote (top left; C. latrans), white-tailed deer (top right; O. virginianus), a great blue heron (bottom left; A. herodias), and a hooded merganser (bottom right; L. cucullatus). (TIFF) [file pone.0310888.s003.tiff]

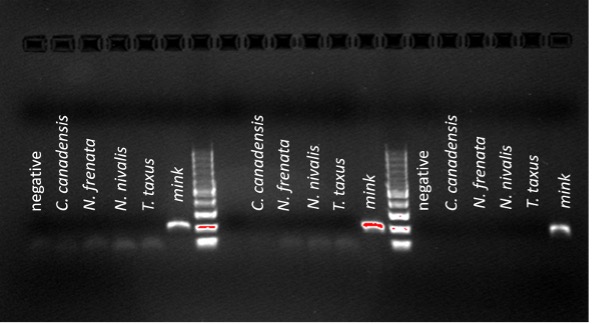

Supplement: S4 Fig — For each group of six wells, left to right is a negative control, DNA from closely related species, and mink. Each set of six columns/wells were run at a different annealing temperature. The left six wells were run at 66.2°C, the middle at 65.6°C, and the right at 65.2°C. The bands highlighted red indicate areas with saturated signal intensity. (JPG) [file pone.0310888.s004.jpg]

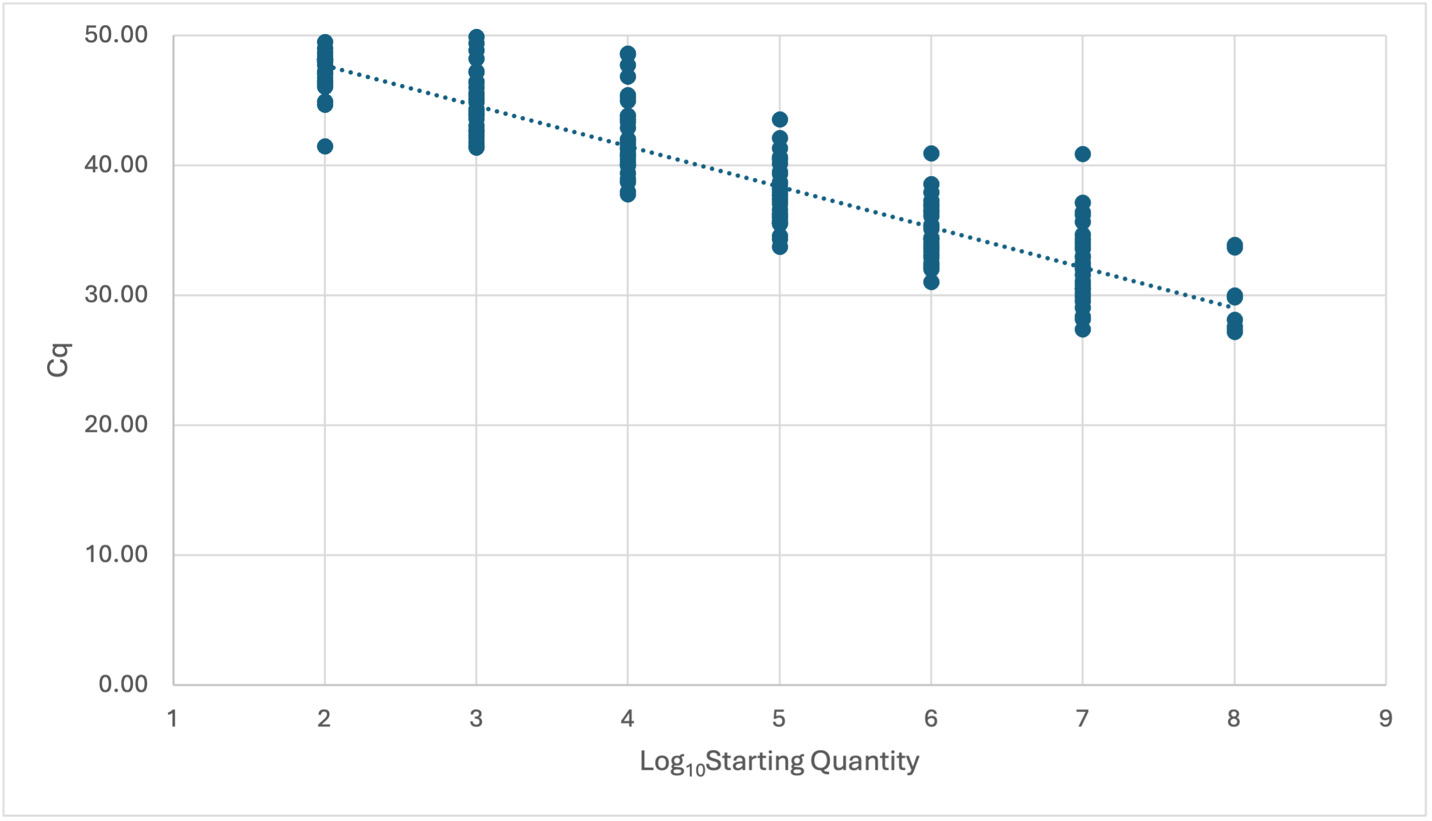
S5 Fig.

Supplement: S5 Fig — There were no detections at or below 1.0x101 copies. (DOCX) [file pone.0310888.s005.docx]

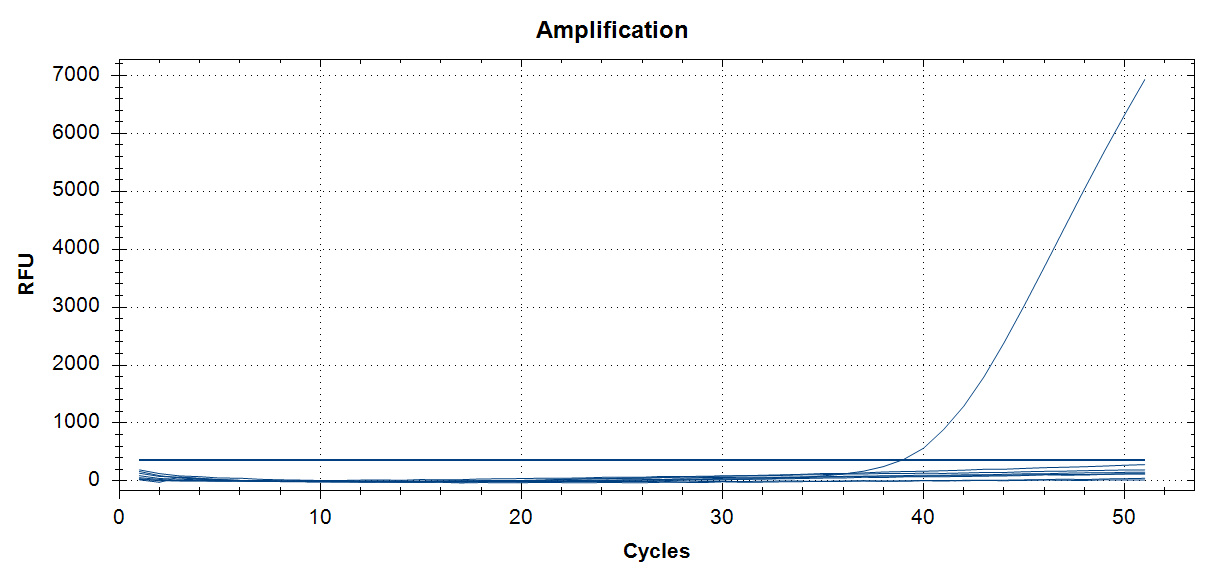


S7 Fig.

Supplement: S7 Fig — Mink amplified at Cq = 38.94 and there was no off-target species amplification within 50 cycles when using an annealing temperature of 65.6°C. (DOCX) [file pone.0310888.s007.docx]

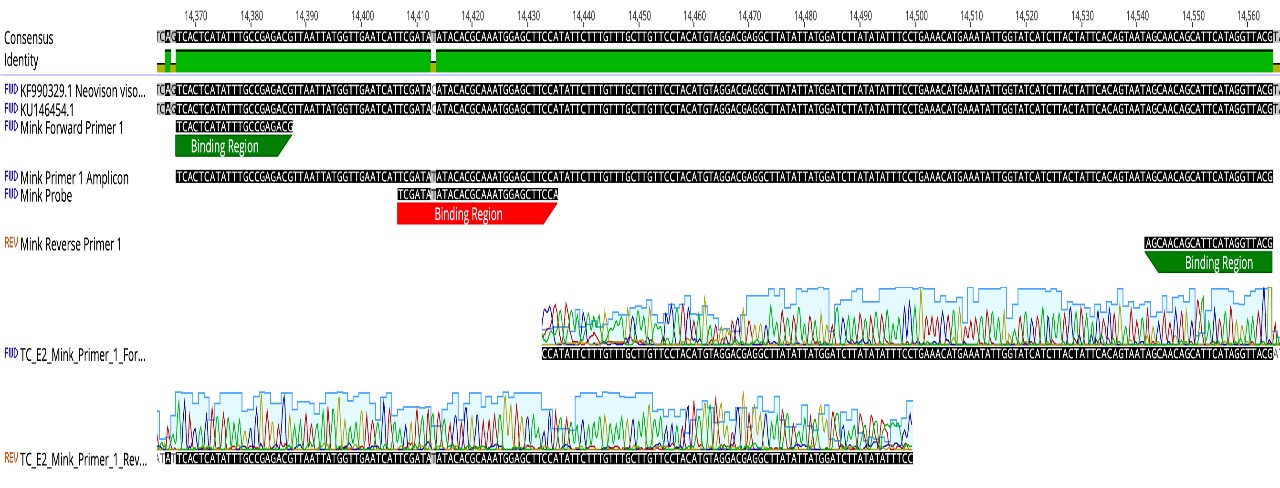

Supplement: S8 Fig — From Tri-County Fish and Wildlife Area in northeastern Indiana. The field sample is aligned with N. vison genome vouchers, cytochrome b (cytb) gene, primer set, probe, and amplicon. (JPG) [file pone.0310888.s008.jpg]
