# Supplementary material for: Comparing the effectiveness of environmental DNA and camera traps for surveying American mink (Neogale vison) in northeastern Indiana
Source: PLoS One. 2024 Sep 23;19(9):e0310888. doi: 10.1371/journal.pone.0310888 (PMC11419345; doi:10.1371/journal.pone.0310888)
Supplement: S6 Fig — Site (CZ = Fort Wayne Children’s Zoo, EM = Eagle Marsh Nature Preserve, GC = Lakeside Golf Course, LC = LC Nature Park, TC = Tri-County Fish and Wildlife Area), survey week, and average Cq values are included. (DOCX) [file pone.0310888.s006.docx]

| Site | Week | C_q_ Values |
| --- | --- | --- |
| CZ | 1 | 33 |
| EM | 3 | 43, 44 |
| GC | 1 | 38, 42, 45 |
| LC | 1 | 41 |
| TC | 1 | 38, 39, 40 |

S6 Fig.
