# Supplementary material for: Comparing the effectiveness of environmental DNA and camera traps for surveying American mink (Neogale vison) in northeastern Indiana
Source: PLoS One. 2024 Sep 23;19(9):e0310888. doi: 10.1371/journal.pone.0310888 (PMC11419345; doi:10.1371/journal.pone.0310888)
Supplement: Attachment — Submitted filename: Rebuttal Letter.docx [file pone.0310888.s010.docx]

Di Girolamo et al. Revisions

Thank you to both the editor(s) and the reviewers for your comments and edits to this manuscript. With your help we have definitely increased the effectiveness and delivery of the article. The following are our responses to the specific edits that the editor(s) and/or the reviewers requested. All minor edits to grammar, spelling, or word choice were revised but were not referred to specifically in this letter.

1. Please ensure that your manuscript meets PLOS ONE's style requirements, including those for file naming. The PLOS ONE style templates can be found at <https://journals.plos.org/plosone/s/file?id=wjVg/PLOSOne_formatting_sample_main_body.pdf> and <https://journals.plos.org/plosone/s/file?id=ba62/PLOSOne_formatting_sample_title_authors_affiliations.pdf>
   a. File was renamed to meet guidelines.
2. In your Methods section, please provide additional information regarding the permits you obtained for the work. Please ensure you have included the full name of the authority that approved the field site access and, if no permits were required, a brief statement explaining why.
   a. Since no animals were handled during this study, we did not require any permits, although we did obtain permissions from landowners for site access. This statement has been added to the Methods section (Line 214-217).
3. We note that the grant information you provided in the ‘Funding Information’ and ‘Financial Disclosure’ sections do not match. When you resubmit, please ensure that you provide the correct grant numbers for the awards you received for your study in the ‘Funding Information’ section.
   a. Grant information provided in “funding information” and “financial disclosure” sections were corrected.
4. We note that you have provided funding information that is not currently declared in your Funding Statement. However, funding information should not appear in the Acknowledgments section or other areas of your manuscript. We will only publish funding information present in the Funding Statement section of the online submission form. Please remove any funding-related text from the manuscript and let us know how you would like to update your Funding Statement. Currently, your Funding Statement reads as follows:
   "We thank the Indiana Department of Natural Resources for funding this study. The funder had no role in study design, data collection and analysis, nor the decision to publish. However, an agent of the funder (G. Albers) contributed to the preparation of the manuscript." Please include your amended statements within your cover letter; we will change the online submission form on your behalf.
   a. We removed funding-related text from manuscript and would like our Funding Statement to say: “This project was funded in part by a Federal Aid in Wildlife Restoration grant (F23AF02947; W-51-R-03) in cooperation with the Indiana Department of Natural Resources, Division of Fish & Wildlife and the U.S. Fish & Wildlife Service, Wildlife and Sport Fish Restoration Program. The funder had no role in study design, data collection and analysis, nor the decision to publish. However, an agent of the funder (G. Albers) contributed to the preparation of the manuscript.” This has been added to our cover letter. Removed funding-related text from Acknowledgments section. (Line 566-571)
5. We note that you have referenced (Albers, Geriann. 2022. Unpublished data. Indiana Department of Natural Resources.
   Indianapolis, IN.) which has currently not yet been accepted for publication. Please remove this from your References and amend this to state in the body of your manuscript: (ie “Bewick et al. [Unpublished]”) as detailed online in our guide for authors
   <http://journals.plos.org/plosone/s/submission-guidelines#loc-reference-style>
   a. We removed the unpublished reference “Albers 2022” from our References. We have added to the body of the manuscript “Albers. [Unpublished]” (Line 67, 596)
6. We note that Figures 1, S2 and S3 in your submission contain copyrighted images. All PLOS content is published under the Creative Commons Attribution License (CC BY 4.0), which means that the manuscript, images, and Supporting Information files will be freely available online, and any third party is permitted to access, download, copy, distribute, and use these materials in any way, even commercially, with proper attribution. For more information, see our copyright guidelines: <http://journals.plos.org/plosone/s/licenses-and-copyright>.
   a. These photos in Figures 1, S2, and S3 were collected by the authors during the project period.

Reviewer 1:

- Line 55. Added “conserve and” (Line 55)
- Line 58. The statement that American mink occurs in low abundance should be updated with information and a short explanation of why this is partly due to male territoriality.
  - Changed “low abundance” to “low density” for clarity. Added a statement about why low densities are partly due to male territoriality. (Line 58, Line 58-60)
- Line 60. Instead of mentioning small vertebrate populations, Authors should mention some important (primary, secondary) food categories.
  - Added a sentence about primary and secondary food sources. (Line 63-64)
- Line 61-65. Among the factors that negatively affect native American mink populations, it could be mentioned here that farm minks may also cause conservation problems in wild mink populations, e.g., by influencing the genetic structure (if this exists in the area).
  - Included information about farm mink affecting wild mink populations. (Line 71-74)
- Line 67-69. Unfortunately, feral American minks can compete not only in Europe, and not only with the European mink. Authors could also cite more general literature here.
  - Added references for feral American minks competing South America and Asia, in addition to Europe. (Line 75)
- Line 81. …. secretive, small or rare species …
  - Added “, small,” (Line 90)
- Line 92. [27, 28],
  - Added “,” (Line 101)
- Line 100. In general, the limitations of the methodology should also be mentioned.
  - Discussed limitations of methodology (Line 127-132)
- Line 105. Padgett-Stewart should be replaced by 21 and further studies/numbers.
  - Replaced “Padgett-Stewart” with “21” and added more literature. (Line 123-124)
- Line 110. Somewhere here, even before the objectives, it should be mentioned which carnivore species or groups this combined method (eDNA + camera trapping) was used for and with what results. The detection effectiveness from simultaneous hair trapping (for DNA analysis) + camera trapping methods could also be mentioned.
  - Mentioned which carnivore species/groups and results. Discussed comparison for hair trapping for DNA analysis and eDNA (Line 109-119)
- Line 116. Unfortunately, this hypothesis needs to be improved. That is why other studies should be mentioned first; it would be easier to formulate a theory based on experiences from studies on terrestrial or semiaquatic species.
  - Revised hypothesis to be more thorough. (Line 139-146)
- Materials and methods
  I recommend inserting a map of the study areas.
  An additional table in the electronic supplementary materials could summarize the main habitat characteristics, making the description clearer.
  - Added brief study site descriptions in text to provide a clearer picture of habitat. We did not include a map in this section as the course detail required to show all sites would make the map useless in regard to showing site details. (Line 164-173)
- Line 205. Erminea
  - Corrected. (Line 249)
- Line 287-288. The part of the sentence in brackets rather fits the methodology.
  - Moved sentence in brackets to Methods (Line 180)
- Line 291. Rather: About two-thirds (64%)…
  - Changed to “two-thirds” (Line 340)
- Line 457. Although it seems insignificant, sparse vegetation cover can also be influential if it is impossible to deploy enough camera traps safely. That can be a problem in the case of systematic camera trapping.
  - Mentioned how sparse vegetation can influence systematic camera trapping. (Line 526-529)
- Line 459. It is unnecessary to highlight the name of Spain here.
  - Removed “Spain” (Line 534)
- Line 462-463. A comparative study should be cited (the ecological guilds of the two mink species are identical).
  - Cited comparative study of both mink species’ ecological guilds (Line 538)
- Line 474. Environmental DNA (at the start of the sentence).
  - Wrote out “Environmental DNA” at the start of the sentence (Line 550)
- In the Discussion section, I propose a more specific formulation of how the obtained results can be used for monitoring semiaquatic carnivores from conservation, research, and management perspectives.
  - Added how these results can be used for monitoring semiaquatic carnivores from various perspectives (Line 541-544)
- I recommend comparing the results obtained with those of others in more detail.
  - Compared results to those of others in more detail throughout the discussion (Line 444-455, 461-480, 488-497, 520, and elsewhere)
- In the end, a sentence could be made about the perspective of the broader application of the combined method.
  - Added sentence at the end of Conclusions, highlighting a broader application of combining the methods (Line 560-564)

Reviewer 2:

- The LOD and LOQ are too high and the basis for their calculation is unclear: the Methods states that the experiments were performed using 10^2-10^6 copies/uL DNA, but the Results state that the LOD is 327 copies/uL and the LOQ is 3270 copies/uL. How were these calculated? Also, in general eDNA studies, LODs of around 1-5 copies/uL are often reported, and 327 copies/uL is too low sensitivity. Is this calculation correct?
  - The standard curve was made from a gBlock sent from IDT. From the gBlock sent, we made serial dilutions from 10^1^ to 10^8^. We determined the number of copies in the original gBlock to be 1.02x10^10^ (this calculation was off, we fixed it in the revision) using the copy number/dilution calculator from ThermoFisher (<https://www.thermofisher.com/us/en/home/brands/thermo-scientific/molecular-biology/molecular-biology-learning-center/molecular-biology-resource-library/thermo-scientific-web-tools/dna-copy-number-calculator.html>). From this, we made serial dilutions of 1:10 until 1.02x10^1^. LoD was set at the minimum concentration that had a detection for at least one of the three replicates and LoQ was set at the minimum concentration that had a detection for all three replicates (Line 287-290). The LoD for this study was 1.02x10^2^ (or 102 copies) because at 1.02x10^1^, we did not have any detections. The LoQ for this study was 1.02x10^3^, where 100% of replicates consistently amplified. LoDs between 1-5 copies/uL is common, indicating this assay may not be as sensitive. We added a few sentences addressing this in the results and discussion (Line 380-384, 487-496).
- In comparing cost calculations, it is not fair as a comparison to include costs for primer testing and methodological refinements in the cost of eDNA. Furthermore, it is also not fair that the cost of eDNA does not include the cost of general consumables, making this comparison meaningless. Either the comparison of costs should be omitted from the paper or a meaningful and fair comparison should be made for the reader.
  - Fixed cost calculations to exclude costs for primer testing and methodological refinements. Included costs for general consumables. Recalculated costs to be individual costs (rather than costs of packs – e.g., calculated cost of 1 filter funnel multiplied by the number of filter funnels used, rather than including cost of the 50 pack of filter funnels we actually bought) and estimated supplies assuming primers/probe and standard curve was figured out, and plate runs were perfect the first time. The cost of supplies used to do extractions, gel electrophoresis, and sequencing of field positives are a rough estimate – will vary based on how many positives are detected. Also changed in-text table to be a summary and an Excel file with itemized list/costs will be included in supplementary material (S9 Fig., Line 423-434)
- The cited literature is geographically biased. Almost all of the cited literature on eDNA research consists of work conducted in North America or Europe. eDNA research has been also developing in Asia, Oceania and other regions. The authors should pay respect to previous studies conducted in those other regions.
  - Added literature from other parts of the world throughout manuscript.
- Raw qPCR data should be disclosed. Disclosure of raw data is essential to assess the validity of the manuscript.
  - Included raw qPCR data for comparison of mink vs other species to show primer/probe specificity (supplementary figure 7, Line 392) and for cumulative standard curve (supplementary figure 5, Line 384), as well as agarose gel showing primer specificity (supplementary figure 4, Line 374). Also included a summary table of field positive samples and their respective Cq values (supplementary figure 6, Line 392). The data used for analysis of detection probabilities and naïve occupancy can be found in Table 1. We can make other data available upon request, but we are not aware of disclosure of raw qPCR data for all plate runs being a standard in the field.
- Line 187 and elsewhere. The general abbreviation for cytchrome b gene is CytB, not MYT-B.
  - Changed “MYT-B” to “*cytb*” (Line 231 and elsewhere)
- Line 207. Amount of template DNA for in vitro check is needed.
  - Added amount of template DNA used. (Line 251)
- Line 210. The in vitro tests were performed with the Ta at 65.6 degrees?
  - Clarified the in vitro tests were ran at an annealing temp. of 65.6 degrees (Line 255)
- Line 231. FAM (probe)" should be "FAM (mink)".
  - Changed “”FAM(probe)” to “FAM(mink)” (Line 281)
- Line 270. This paragraph should be under an independent section.
  - Moved to an independent section. (Line 321)
- Line 340. Gel electrophoresis never amplifies DNA.
  - Changed wording. (Line 393)
- Line 342. "Off target species" should read "Closest off-target species in the BLAST test".
  - Changed to “closest off-target species in the BLAST test” (Line 396)
