# Supplementary material for: Comparing the effectiveness of environmental DNA and camera traps for surveying American mink (Neogale vison) in northeastern Indiana
Source: PLoS One. 2024 Sep 23;19(9):e0310888. doi: 10.1371/journal.pone.0310888 (PMC11419345; doi:10.1371/journal.pone.0310888)
Supplement: Attachment — Submitted filename: Response to Reviewers.docx [file pone.0310888.s011.docx]

Di Girolamo et al. Revisions

1. Please ensure that your manuscript meets PLOS ONE's style requirements, including those for file naming. The PLOS ONE style templates can be found at <https://journals.plos.org/plosone/s/file?id=wjVg/PLOSOne_formatting_sample_main_body.pdf> and <https://journals.plos.org/plosone/s/file?id=ba62/PLOSOne_formatting_sample_title_authors_affiliations.pdf>
   a. Fixed title author affiliations to match sample. Changed body formatting to be single-spaced and fixed level 2 headings to be 16pt font. Fixed figure headings (both figures and supplementary figures) to match correct formatting and fixed file formatting/names.
2. In your Methods section, please provide additional information regarding the permits you obtained for the work. Please ensure you have included the full name of the authority that approved the field site access and, if no permits were required, a brief statement explaining why.
   a. Since no animals were handled during this study, we did not require any permits, although we did obtain permissions from landowners for site access. This statement has been added to the Methods section (Lines 219-222).
3. We note that your Data Availability Statement is currently as follows: All relevant data are within the manuscript and its supporting information files.

Please confirm at this time whether or not your submission contains all raw data required to replicate the results of your study. Authors must share the “minimal data set” for their submission. PLOS defines the minimal data set to consist of the data required to replicate all study findings reported in the article, as well as related metadata and methods (<https://journals.plos.org/plosone/s/data-availability#loc-minimal-data-set-definition>).

a. We confirm that our submission contains all raw data required to replicate the results of our study.

4. Please review your reference list to ensure that it is complete and correct. If you have cited papers that have been retracted, please include the rationale for doing so in the manuscript text, or remove these references and replace them with relevant current references. Any changes to the reference list should be mentioned in the rebuttal letter that accompanies your revised manuscript. If you need to cite a retracted article, indicate the article’s retracted status in the References list and also include a citation and full reference for the retraction notice.
   a. We checked the references list and everything is correct.

From the reviewers:

Reviewer 1:

- I have doubts about the calculation of gBlock copy numbers. In my experience, if the qPCR efficiency is high enough (97.5% in this study), the Ct value for 100 copies per PCR reaction would be around 35. But in this study, the Ct values for 100 copies are much higher than 40, over 45. This is unbelievable in my experience. I suggest the authors to check the copy number of the gBlock again and again.
  - On August 30^th^, 2024, we rechecked our calculations for the gBlock and the serial dilutions. The gBlock ordered was a 250ng desiccate of a 198bp fragment with a molecular weight of 122189.4. It was diluted to 10ng/μL, per the instructions provided by IDT, by adding 25μL of TE pH 8.0, which we calculated to be equivalent to 4.92x10^10^ DNA copies. From that, we made 200μL of 1.0x10^9^ by adding 4.0μL of 10ng/μL to 196μL of TE. We then made serial dilutions from the 1.0x10^9^ DNA copies down to 1.02x10^1^ DNA copies. We reran our old standard curve, as well as remade the dilutions and ran those dilutions to double check. Our results were consistent as the original manuscript, with the LoD was 1.0x10^2^ and LoQ was 1.0x10^3^, still with C_t_ values up to almost 46. At 1.0x10^1^, we did not have any detections.
- I also have a question about Fig. S5. It is described that three replicates were used for LOD and LOQ tests. But there are many points in S5 Fig. If the authors have combined the data from different experiments, it is not a good way.
  - Figure 5 was a cumulative standard curve of all standard curves ran on each plate throughout the study. We ran 3-replicates for each dilution for the standard curve ranging from 1.0x10^2^ to 1.0x10^6^. LOD and LOQ were determined from the results of all standard curves ran throughout the study. We can remove this figure, if preferred.
- Finally, the amount of DNA (in ng or pg), not the volume, should be described for the specificity test.
  - We changed the volume of DNA to an amount of DNA in line 404.

Reviewer 2:

- Conclusions, Lines 551-552. Please make this sentence consistent with the results.
  - Sentences in Lines 551-554 (now lines 574-577) were fixed to be consistent with results.
